# The Histone Demethylase KDM3B Promotes Osteo-/Odontogenic Differentiation, Cell Proliferation, and Migration Potential of Stem Cells from the Apical Papilla

**DOI:** 10.1155/2020/8881021

**Published:** 2020-10-07

**Authors:** Chen Zhang, Xiao Han, Yuncun Liang, Huina Liu, Zhipeng Fan, Jianpeng Zhang

**Affiliations:** ^1^Laboratory of Molecular Signaling and Stem Cells Therapy, Beijing Key Laboratory of Tooth Regeneration and Function Reconstruction, School of Stomatology, Capital Medical University, 100050, China; ^2^Department of Endodontics, Beijing Stomatological Hospital, School of Stomatology, Capital Medical University, 100050, China

## Abstract

Understanding the regulation mechanisms of mesenchymal stem cells (MSCs) can assist in tissue regeneration. The histone demethylase (KDM) family has a crucial role in differentiation and cell proliferation of MSCs, while the function of KDM3B in MSCs is not well understood. In this study, we used the stem cells from the apical papilla (SCAPs) to test whether KDM3B could regulate the function of MSCs. By an alkaline phosphatase (ALP) activity assay, Alizarin red staining, real-time RT-PCR, and western blot analysis, we found that KDM3B enhanced the ALP activity and mineralization of SCAPs and promoted the expression of runt-related transcription factor 2 (RUNX2), osterix (OSX), dentin sialophosphoprotein (DSPP), and osteocalcin (OCN). Additionally, the CFSE, CCK-8, and flow cytometry assays revealed that KDM3B improved cell proliferation by accelerating cell cycle transition from the G1 to S phase. Scratch and transwell migration assays displayed that KDM3B promoted the migration potential of SCAPs. Mechanically, microarray results displayed that 98 genes were upregulated, including *STAT1*, *CCND1*, and *FGF5*, and 48 genes were downregulated after KDM3B overexpression. Besides, we found that the Toll-like receptor and JAK-STAT signaling pathway may be involved in the regulating function of KDM3B in SCAPs. In brief, we discovered that KDM3B promoted the osteo-/odontogenic differentiation, cell proliferation, and migration potential of SCAPs and provided a novel target and theoretical basis for regenerative medicine.

## 1. Introduction

With the development of the technique, the treatment outcome of tooth loss and orofacial bone defects is improved continuously. Nonetheless, the limited bone intrinsic regenerative ability or the impaired host regenerative ability will affect the tissue repair and regeneration [1]. In recent years, stem cell-based therapy is a prospecting strategy for repairing and regenerating dental and orofacial bone defects [2]. Mesenchymal stem cells (MSCs) are attractive candidates for tissue regeneration due to their capacity for self-renewal and multiple differentiation potentials [3, 4]. At present, stem cells are divided into odontogenic stem cells including the stem cells from the apical papilla (SCAPs), stem cells from the dental pulp (DPSCs) and periodontal ligament (PDLSCs), and nonodontogenic stem cells including bone marrow stem cells (BMSCs) [5, 6]. However, the underlying molecular mechanism of MSCs remains to be unveiled before widespread clinical application in dental and orofacial bone regeneration.

Histone methylation is a major regulator of epigenetic modification, which could be critical for determining the cell fate of MSCs [7, 8]. The status and location of lysine methylation determine that it acts as an activator or suppressor of transcription, such as H3K4, H3K36, and H3K79 methylation activating transcription and H3K9, H3K27, and H4K20 methylation suppressing transcription, and is regulated by histone methyltransferases and demethylases [8, 9]. Therefore, the function and balance between histone methyltransferases and demethylases determine gene expression. The largest family of histone demethylase (KDMs) has the Jumonic (JmjC) domain, including the KDM2-7 subfamily; these histone demethylases are thought to have functions in maintaining cell fate and genome stability [10, 11]. At present, histone demethylation is an important epigenetic mechanism in dental and orofacial bone regeneration [12–14]. And mounting evidence shows that the KDM subfamily may be linked to osteo-/odontogenic differentiation and cell proliferation [12, 15–21].

The current results reflected that the loss of H3K9me2 is associated with the activation of gene expression [22]. Lysine-specific demethylase 3B (KDM3B), also known as JMJD1B, which is a JmjC domain-containing protein, was regarded as a lysine demethylase for H3K9 demethylation (H3K9me2) [22, 23]. Moreover, KDM3B is responsible for the demethylation of H3K9me2 which has been shown to change the state of chromatin modification and dynamically regulate the gene expression of differentiated cells and regulate the proliferation of cells [19, 24–26]. Subsequently, researchers confirm that KDM3B is also important for the erasure of H4R3me2s which is also necessary for cellular processes including osteogenic differentiation and cell cycle potential of MSCs [22, 27], while the molecular mechanisms by which KDM3B regulates osteo/-odontogenic differentiation and self-renewal capacity in MSCs have not been well characterized and need to be explored.

In this study, our team aims to explore the potential function and mechanism of the KDM3B in MSCs by using SCAPs. Our findings show that the KDM3B promotes the osteo-/odontogenic differentiation, cell proliferation, and migration potential in human SCAPs. Moreover, the candidate targets of KDM3B are identified and provide the novel target and theoretical basis for tissue regeneration.

## 2. Materials and Methods

### 2.1. Cell Cultures

Apical papilla stem cells (SCAPs) were used in these experiments following the ISSCR “Guidelines for the Conduct of Human Embryonic Stem Cell Research,” after obtaining patient consent, and the study was approved by the ethics committee of Beijing Stomatological Hospital, Capital Medical University. SCAPs were obtained as previously depicted [28, 29]. Briefly, the apical tissue was peeled off rapidly after wisdom tooth extraction and digested with 3 mg/mL type I collagenase (Worthington Biochemical Corp., Lakewood, NJ, USA) and 4 mg/mL Dispase (Roche Diagnostics Corp., Indianapolis, IN, USA) at 37°C for 45 min. In our previous study, we described the culture of SCAPs [28, 29]. The cell images under the microscope are shown in Figure S1.

### 2.2. Plasmid Construction and Viral Infection

Construction of plasmids following standard techniques was confirmed using restriction enzyme digestion analysis or sequencing. A hemagglutinin (HA) tag combined with the full-length sequence of *KDM3B* was subcloned into the pQCXIN retroviral vector by the BamH1 and AgeI restriction sites. Short hairpin RNA (shRNA) of *KDM3B* was subcloned into the pLKO.1 lentiviral vector (Addgene). The scramble shRNA (Scramsh) was purchased from Addgene. The target sequence for the shRNA of *KDM3B* was 5′-AGGCACATTACATTTAGTC-3′.

### 2.3. Alkaline Phosphatase (ALP) and Alizarin Red Detection

SCAPs were cultured in the osteogenesis differentiation medium for 3 days, and ALP activity was detected with an ALP activity kit (Sigma-Aldrich, St. Louis, MO, USA). Cells were cultured in osteogenesis differentiation medium for 2 weeks and then stained with Alizarin red according to the manufacturer's instructions, as described in our previous work [15].

### 2.4. Real-Time Reverse Transcriptase Polymerase Chain Reaction (Real-Time RT-PCR)

The extraction of total RNA of SCAPs, the synthesis of cDNA, and the reactions of real-time RT-PCR were tested as described in our previous study [30]. By using the method of 2^-*△△*CT^, the expression of genes was determined and normalized based on *GAPDH*. The primers used in this study are listed in Table 1.

### 2.5. Western Blot Analysis

25 *μ*g protein isolated from SCAPs was diluted in PBS to a total volume of 25 *μ*L. The western blot was performed as described in our previous study [30]. The primary antibodies used in the study are as follows: KDM3B antibody (cat no. 19915-1-AP, Rabbit Polyclonal, USA), RUNX2 antibody (cat no. ab76956, Abcam), OSX antibody (cat no. ab209484, Abcam), OCN antibody (cat no. bs-4917R, Bioss, China), DSPP antibody (cat no. bs10316R, Bioss, China), and histone H3 antibody (cat no. SC-56616, Santa Cruz Biotechnology, Santa Cruz, CA, USA).

### 2.6. CFSE Assay

To detect the proliferation ability of SCAPs, carboxyfluorescein succinimidyl ester (CFSE) assays were applied as previously described [30]. The proliferation ability of SCAPs was tested by flow cytometry (FACSCalibur, BD Biosciences, USA) after staining with CFSE reagents. The results were documented using flow cytometry with a 488 nm laser. ModFit LT (Verity Software House, Topsham, ME, USA) was applied to calculate the proliferation index.

### 2.7. CCK-8 Assay

To further test the proliferation ability of SCAPs, the Cell Counting Kit-8 assay (Dojindo, Kumamoto, Japan) was performed as previously described [30]. 7 × 10^3^ cells/well were seeded into 96-well plates. After 24 h and 48 h incubation, the culture medium was replaced with 10 *μ*L CCK-8 reagent mixed with 100 *μ*L DMEM. 2 h of incubation later, the absorbance (OD) was measured with a multiwell spectrophotometer at 450 nm.

### 2.8. Flow Cytometry for the Cell Cycle

The flow cytometry was applied for detecting the cell cycle. As previously described [30], SCAPs (1 × 10^6^) were placed in anhydrous ethanol at 4°C overnight and then incubated with the mixture of propidium iodide (50 *μ*g/mL, Sigma) and 10 *μ*g/mL RNase in the dark for 30 minutes. Cell cycle fractions (G0/G1 phase, S phase, and G2/M phase) were calculated by flow cytometry. PI = ((S + G2)/M)/((G0/G1) + S + G2M) was applied to calculate the proliferation index.

### 2.9. Scratch Migration Assays

SCAPs were seeded in 6-well culture plates with 4 × 10^5^ cells per well and cultured in serum-free medium for 48 h. Subsequently, the cells were scratched with a 1000 *μ*L pipet tip (Axygen® Corning, NY, USA) to create a wound and washed twice with PBS to clear the floating cells and then continuously incubated in the fresh culture medium. Scratch images were observed under a microscope at 0 h, 24 h, and 48 h after wound scratch, the degree of wound closure was tested, and the relative width (RW) was calculated by the program of Image-Pro 1.49v (National Institutes of Health, USA).

### 2.10. Transwell Chemotaxis Assays

To detect the chemotaxis ability of SCAPs, 24-well chemotaxis chambers were applied for this experiment. SCAPs suspended in 100 *μ*L serum-free medium (Invitrogen, Carlsbad, CA, USA) were placed in the upper chamber at a density of 2 × 10^4^ cells, while 600 *μ*L of serum-free medium with the addition of 15% fetal bovine serum (FBS) was added in the bottom chamber. The chemotactic ability of the cells will cause the cells in the upper chamber to migrate through the hole to the lower chamber. After 24 and 48 hours, 10 visual fields were randomly selected for counting transferred cell numbers using a microscope (Olympus, Japan).

### 2.11. Microarray Analysis

The gene expression profile was performed and analyzed by using the Human Gene 1.0 ST Array (Affymetrix, USA). Abiding by the methods as described previously [30], differentially expressed genes were analyzed using the Affymetrix GeneChip Operating Software (Affymetrix, USA). The selected threshold set for differentially mRNAs was a fold change > 1.5 and a *P* value < 0.05.

### 2.12. Statistics

Each experiment was done at least in triplicate. All the data were analyzed by the SPSS17 statistical software (SPSS Inc., Chicago, IL, USA). Significance was determined using Student's *t*-test. *P* ≤ 0.05 was regarded as statistically significant.

## 3. Results

### 3.1. KDM3B Increased the Osteo-/Odontogenic Differentiation Potential of SCAPs

**Figure 1:**
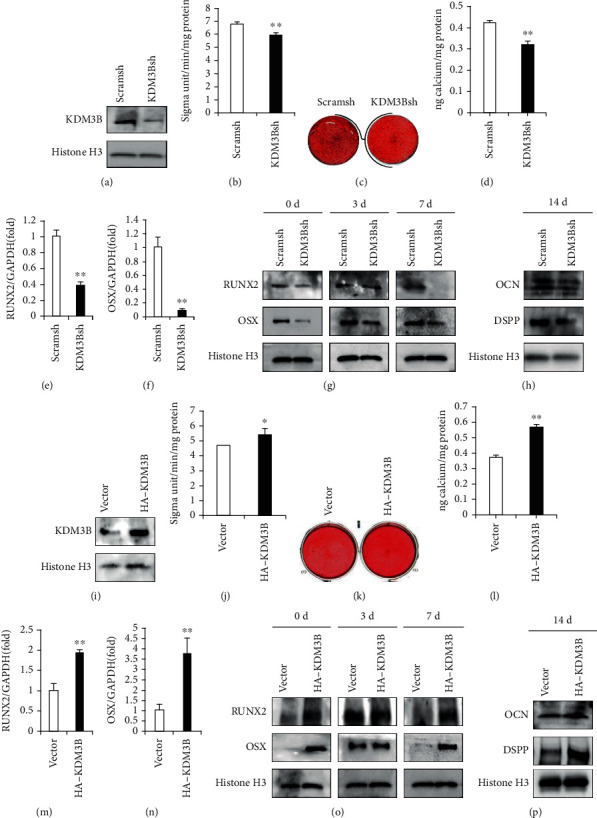
KDM3B enhanced the osteo-/odontogenic differentiation potential of SCAPs. (a) The knockdown efficiency of KDM3B in SCAPs was tested by western blot. (b) KDM3B knockdown significantly depressed the ALP activity in SCAPs. (c) The Alizarin red staining and (d) the quantitative calcium analysis showed that KDM3B knockdown reduced the mineralization capacity of SCAPs compared with the control group. (e, f) Real-time RT-PCR analysis confirmed that KDM3B knockdown reduced the expression of (e) *RUNX2* and (f) *OSX* in SCAPs. (g) Western blot analysis showed the expression of RUNX2 and OSX in the KDM3B knockdown group and the control group. Histone H3 served as an internal control. (h) Western blot analysis revealed that the expression of DSPP and OCN was decreased after KDM3B was knocked down. Histone H3 served as an internal control. (i) The KDM3B overexpression was tested by western blot. (j) KDM3B overexpression significantly enhanced the ALP activity in SCAPs. (k, l) The results of (k) the Alizarin red staining and (l) the quantitative calcium analysis revealed that KDM3B overexpression enhanced the mineralization capacity of SCAPs compared with the control group. (m, n) Real-time RT-PCR analysis revealed that KDM3B overexpression increased the expression of (m) *RUNX2* and (n) *OSX* in SCAPs. (o) Western blot analysis showed the expression of RUNX2 and OSX in the KDM3B overexpression group and the control group. Histone H3 served as an internal control. (p) Western blot analysis revealed that the expression of OCN and DSPP was enhanced after KDM3B was overexpressed. Histone H3 served as an internal control. Statistical significance was determined using Student's *t*-test. All error bars represent SD (*n* = 3). *P* < 0.05. ^∗∗^*P* ≤ 0.01.

To identify the potential roles of KDM3B, we knock down KDM3B in SCAPs through lentiviral transfection. The knockdown efficiency of KDM3B in SCAPs was tested by western blot analysis after 3 days of treatment of 2 *μ*g/mL puromycin (Figure 1(a)). At 3 days after osteo-/odontogenic induction, we found that KDM3B knockdown significantly depressed the ALP activity (Figure 1(b)). The Alizarin red staining and the quantitative calcium analysis were assessed after 2 weeks of in vitro culturing in osteo-/odontogenic induction medium. The results reflected that KDM3B knockdown reduced the mineralization capacity of SCAPs (Figures 1(c) and 1(d)). Real-time RT-PCR analysis confirmed that KDM3B knockdown reduced the expression of *RUNX2* and *OSX* (Figures 1(e) and 1(f)). After osteo-/odontogenic induction, western blot analysis showed downregulated RUNX2 and OSX in the KDM3B knockdown group compared with the control group at 0 and 7 days (Figure 1(g)). Moreover, we detected the osteo-/odontogenic marker proteins at 2 weeks after osteo-/odontogenic induction, and the western blot results reflected that expression of OCN and DSPP was decreased after KDM3B was knocked down in SCAPs (Figure 1(h)). To further investigate the osteo-/odontogenic differentiation function of KDM3B in SCAPs, the HA-KDM3B sequence was inserted into the retroviral vector which was used to infect SCAPs. The KDM3B overexpression was tested by western blot (Figure 1(i)). At 3 days after osteo-/odontogenic induction, we discovered that KDM3B overexpression significantly enhanced the ALP activity (Figure 1(j)). At 2 weeks after osteo-/odontogenic induction, the Alizarin red staining and the quantitative calcium analysis revealed that KDM3B overexpression enhanced the mineralization capacity of SCAPs (Figures 1(k) and 1(l)). Real-time RT-PCR analysis confirmed that KDM3B overexpression promoted the expression of *RUNX2* and *OSX* (Figures 1(m) and 1(n)). After osteo-/odontogenic induction, western blot analysis showed upregulated RUNX2 and OSX in the KDM3B overexpression group compared with the control group at 0 and 7 days (Figure 1(o)). In parallel, after 2 weeks of osteo-/odontogenic induction, the western blot results revealed that the expression of OCN and DSPP was enhanced after KDM3B was overexpressed (Figure 1(p)).

### 3.2. KDM3B Enhanced the Proliferation Ability of SCAPs

**Figure 2:**
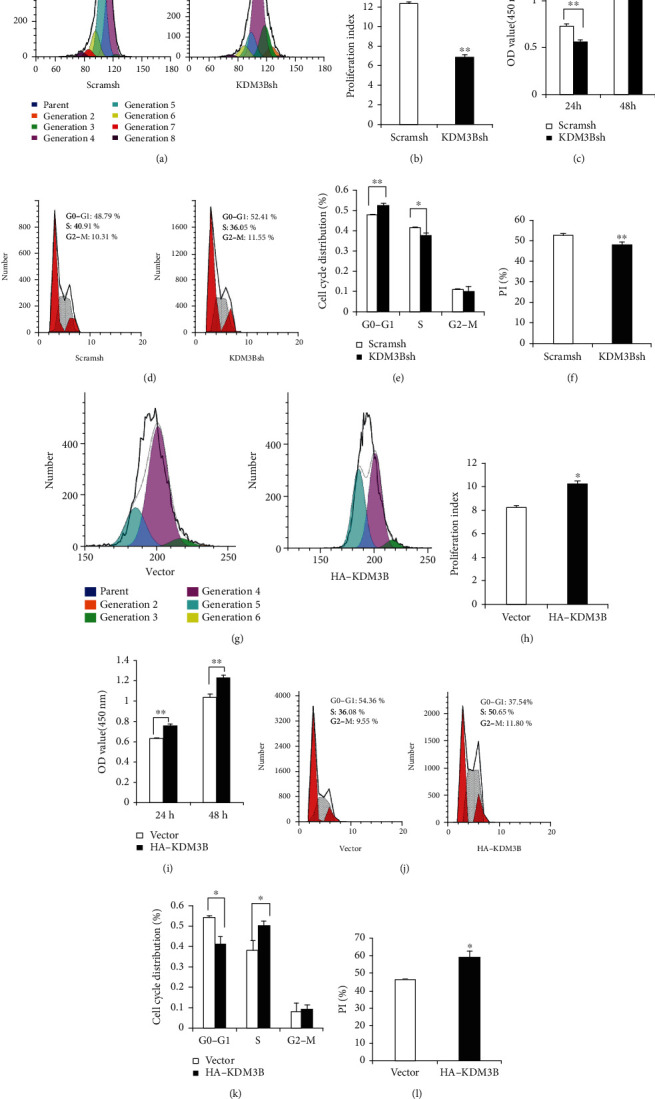
KDM3B enhanced the cell proliferation of SCAPs and regulated the cell cycle. The (a) CFSE assay and (b) quantitative analysis results revealed that KDM3B knockdown decreased the cell numbers at 3 days in SCAPs. (c) The CCK-8 assay revealed that KDM3B knockdown reduced the cell numbers at 24 h and 48 h in SCAPs. (d) Flow cytometric cell cycle analysis revealed that KDM3B knockdown increased the proportion of cells in the G0/G1 phase and decreased the proportion of cells in the S phase. (e) Comparison of cell cycle distribution between the KDM3B knockout group and the control group. (f) The proliferation index between the KDM3B knockout group and the control group. The (g) CFSE assay and (h) quantitative analysis results revealed that KDM3B overexpression enhanced the cell numbers at 3 days in SCAPs. (i) The CCK-8 assay revealed that KDM3B overexpression increased the cell numbers at 24 h and 48 h in SCAPs. (j) Flow cytometric cell cycle analysis revealed that KDM3B overexpression decreased the percentage of cells in the G0/G1 phase and increased the proportion of cells in the S phase. (k) Comparison of cell cycle distribution between the KDM3B overexpression group and the control group. (l) The proliferation index between the KDM3B overexpression group and the control group. Statistical significance was determined using Student's *t*-test. All error bars represent SD (*n* = 3 or 6). *P* < 0.05. ^∗∗^*P* ≤ 0.01.

To detect the effects of KDM3B on cell proliferation potential of SCAPs, the CFSE assay, CCK-8 assay, and flow cytometric cell cycle analysis were performed. After culturing for 3 days, the result of the CFSE assay confirmed that the proliferation potential of SCAPs was suppressed following KDM3B knockdown (Figures 2(a) and 2(b)). The result of the CCK-8 assay showed that cell numbers in the KDM3B knockdown group were significantly reduced compared with those in the control group at 24 h and 48 h (Figure 2(c)). Then, after culturing for 3 days, flow cytometric cell cycle analysis was carried out. The results revealed that KDM3B knockdown increased the proportion of cells in the G0/G1 phase and decreased the percentage of cells in S phases (Figures 2(d) and 2(e)). After calculation, the proliferation index was significantly lower in the KDM3B knockdown group than in the control group (Figure 2(f)). To further prove whether KDM3B could affect the proliferation potential of SCAPs, the CFSE assay, CCK-8 assay, and flow cytometric cell cycle analysis were performed in KDM3B overexpression SCAPs. After culturing for 3 days, the CFSE assay showed that the proliferation potential of SCAPs was enhanced in the KDM3B overexpression group compared with the control group (Figures 2(g) and 2(h)). The result of the CCK-8 assay showed that cell numbers in the KDM3B overexpression group were significantly increased compared with those in the control group at 24 h and 48 h (Figure 2(i)). Also, after culturing for 3 days, flow cytometric cell cycle analysis was carried out in SCAPs. The results showed that KDM3B overexpression decreased the proportion of cells in the G0/G1 phase and increased the proportion of cells in the S phase (Figures 2(j) and 2(k)). Consistently, the proliferation index was higher in the KDM3B overexpression group than in the control group (Figure 2(l)).

### 3.3. KDM3B Enhanced the Migration and Chemotaxis Potential of SCAPs

**Figure 3:**
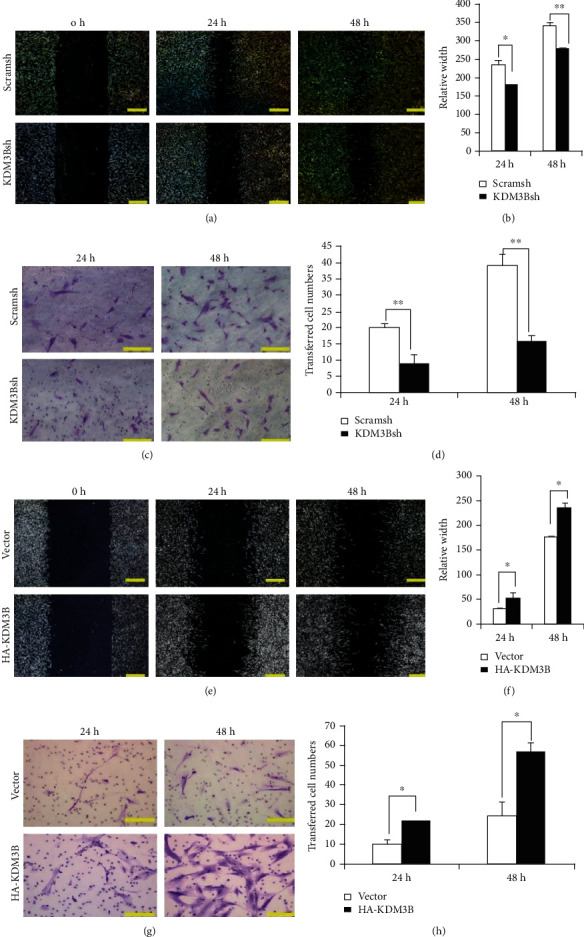
KDM3B enhanced the migration and chemotaxis potential of SCAPs. The (a) scratch migration assay and (b) quantitative analysis results demonstrated that KDM3B knockdown led to a significant decrease in the migration ability of SCAPs at 24 h and 48 h. The (c) transwell chemotaxis assay and (d) quantitative analysis results showed that KDM3B knockdown led to a decrease in the chemotaxis ability of SCAPs at 24 h and 48 h. The (e) scratch migration assay and (f) quantitative analysis results demonstrated that KDM3B overexpression led to a significant increase in the migration ability of SCAPs at 24 h and 48 h. The (g) transwell chemotaxis assay and (h) quantitative analysis results showed that KDM3B overexpression led to an increase in the chemotaxis ability of SCAPs at 24 h and 48 h. Statistical significance was determined using Student's *t*-test. All error bars represent SD (*n* = 3 or 6). *P* < 0.05. ^∗∗^*P* ≤ 0.01.

To test whether KDM3B could regulate the migration potential of SCAPs, the scratch migration and transwell chemotaxis assays were performed. Scratch migration assays demonstrated that KDM3B knockdown led to a significant decrease in the migration ability of SCAPs at 24 h and 48 h (Figures 3(a) and 3(b)). Similarly, the results of the transwell chemotaxis assay confirmed that KDM3B knockdown led to a decrease in the chemotaxis ability of SCAPs at 24 h and 48 h (Figures 3(c) and 3(d)). To further confirm the function of KDM3B in the regulation of the migration of SCAPs, the scratch migration and transwell chemotaxis assays were performed in KDM3B overexpression SCAPs. Scratch migration assay results revealed that KDM3B overexpression led to a significant increase in the migration ability of SCAPs at 24 h and 48 h (Figures 3(e) and 3(f)). The results of the transwell chemotaxis assay confirmed that KDM3B overexpression led to a significant increase in the chemotaxis ability of SCAPs at 24 h and 48 h (Figures 3(g) and 3(h)).

### 3.4. Differentially Expressed Genes and Bioinformatic Analysis in KDM3B overexpression SCAPs

Subsequently, differentially expressed genes between KDM3B overexpression SCAPs and the control group were identified by microarray analysis. The microarray analysis results suggested that there were 146 differential genes in the KDM3B overexpression SCAPs compared with the control group. In these differentially expressed genes, 98 genes were upregulated while 48 genes were downregulated in the KDM3B overexpression SCAPs compared with the control group (Table S1). The upregulated genes include *TGM2*, *ITGA2*, *STAT1*, *CCND1*, *FGF5*, and *SEMA3A*, while the downregulated genes include *COL3A1*, *C5AR2*, and *SERPINF1*.

**Figure 4:**
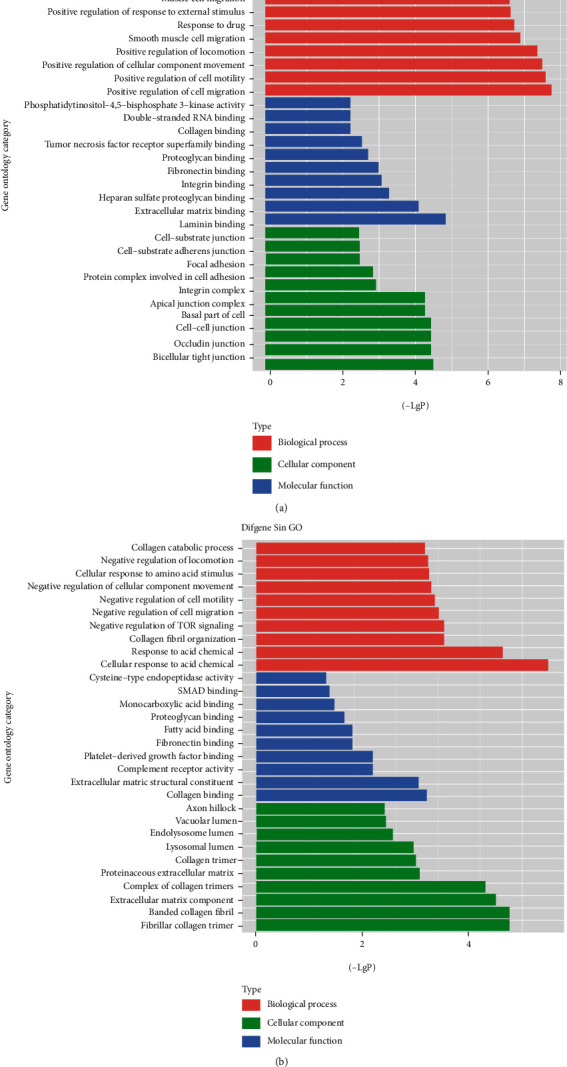
Significant gene ontology (GO) analyses of differentially expressed genes in KDM3B overexpression SCAPs compared with the control group. (a) The significant GO functions of upregulated genes during KDM3B overexpression. (b) The significant GO functions of downregulated genes during KDM3B overexpression. The *y*-axis represents the GO category, and the *x*-axis represents the negative logarithm of the *P* value (−LgP). A larger −LgP indicated a smaller *P* value for the difference.

Then, gene ontology (GO) analysis and pathway analysis were carried out. First, GO analysis was used to identify a statistical enrichment of various biological functions and pathways from the differentially expressed genes in the HA-KDM3B group. By GO analysis, the upregulated GO functions included positive regulation of cell migration, positive regulation of cell motility, positive regulation of cellular component movement, and positive regulation of response to external stimulus (Figure 4(a)). Some important downregulated GO functions included negative regulation of TOR signaling and negative regulation of cell migration (Figure 4(b)).

**Figure 5:**
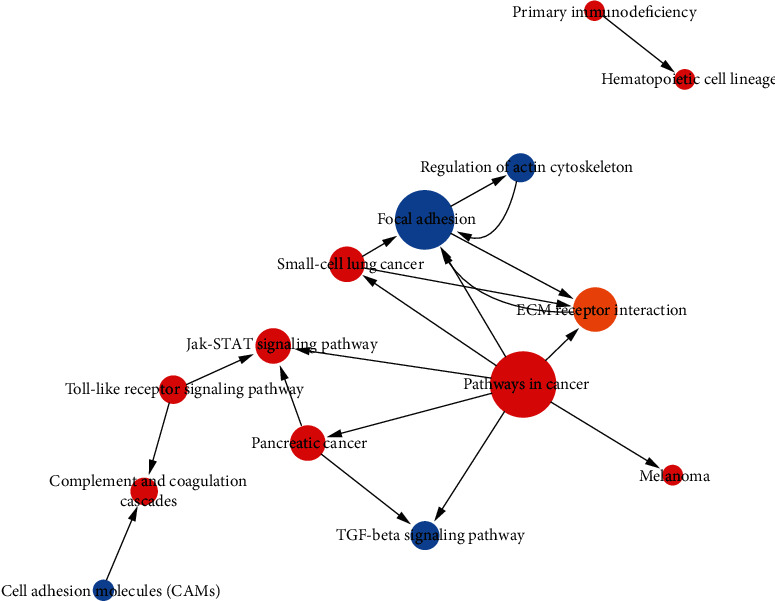
Analysis of the interaction network of significant pathways (Path-net) in the KDM3B overexpression group compared with the control group. A pathway of a high degree means that it played an essential role in the signaling network. Red indicates upregulated pathways, blue indicates downregulated pathways, and yellow indicates up- and downregulated pathways. Circle nodes represent a pathway, and the lines indicate the interactions between two pathways.

We then used the KEGG database to enrich the pathways of significant alterations in differentially expressed genes and identified the pathways of significant changes associated with differentially expressed genes which may play an important role in function regulation of KDM3B (Table S2). After analysis, the Toll-like receptor and the JAK-STAT signaling pathway associated with upregulated genes as well as focal adhesion and the TGF-beta signaling pathway associated with downregulated genes were identified (Figure 5, Table S3).

**Figure 6:**
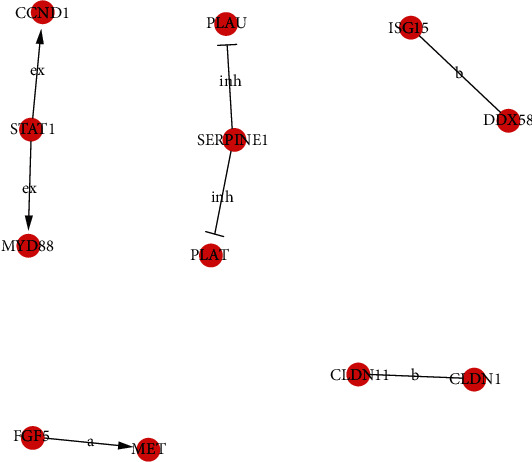
Analysis of the interaction network of differentially expressed genes (Signal-net) in the KDM3B overexpression group compared with the control group. In the Signal-net, the intermediate centrality of genes represents the characteristics of genes. Red represents an important upregulated gene, and the degree of interaction between genes is indicated by the circle size and the interaction is indicated by the line.

To further examine the global network, we calculated connectivity of each gene in the network and determined the node with the highest connectivity by Signal-net analysis. Through the analysis of the significantly regulated GOs and pathways, we found 12 core genes (*CCND1*, *CLDN1*, *CLDN11*, *DDX58*, *FGF5*, *ISG15*, *MET*, *MYD88*, *PLAT*, *PLAU*, and *SERPINE1*) in the KDM3B overexpression group compared with the control group according to the degree of gene interaction (Figure 6, Table S4).

## 4. Discussion

Dental and orofacial bone regeneration depends on the MSCs [31]. Under the condition of osteogenesis, dental tissue-derived MSCs expressed typical osteoblastic markers such as mineralized matrix nodules and differentiate into an osteoblastic/odontoblastic lineage [32]. The maintenance of self-renewal and differentiation potential is essential in MSC-based therapy, but expansion in vitro may lead to the loss of these characteristics [32]. Therefore, improving the function of MSCs will effectively improve the therapeutic efficacy of MSCs.

ALP activity is an early marker of osteo-/odontogenic differentiation. In our study, we proved that KDM3B promotes osteo-/odontogenic differentiation by increasing the expression of ALP. *In vitro* mineralization represents a late marker of osteogenesis. The results of Alizarin red staining and the quantitative calcium analysis revealed that KDM3B enhanced the mineralization in SCAPs. These results all consistent with the data reported in the literature revealed that KDM3B is the key factor of osteogenic differentiation in MSCs [33]. In our study, we found that KDM3B promotes the gene expressions of SCAPs which are involved in the process of cell differentiation. These genes, including *RUNX2* and *OSX*, have been identified as key transcription factors in the early stage of osteo-/odontogenic differentiation [34, 35]. Besides, KDM3B also promoted the expressions of OCN and DSPP in SCAPs. These proteins represent a commitment to differentiate into an osteogenic cell lineage [36–38]. The above results suggested that KDM3B is a potential enhancer of osteo-/odontogenic differentiation in SCAPs.

Moreover, new bone formation and graft integration rely on the recruitment of stem cells to target sites and obtained more stem cells for successful directed differentiation in the microenvironment [39]. In the present study, we also confirmed that KDM3B enhanced the proliferation ability of SCAPs by promoting the rapid transition from the G1 phase to the S phase. We also discovered that KDM3B enhanced the migration and chemotaxis ability of SCAPs. This is consistent with the previous report in B cells and liver cells [19, 25]. Combined together, these results suggest that KDM3B has a positive role for the recruitment of SCAPs and facilitates cell expansion.

Previously, Li et al. found that KDM3B demethylates H4R3me2s and H3K9me2 in different promoter regions to facilitate gene expression for the development of hematopoietic stem cells [22]. To further explore its regulatory mechanism, we carried out microarray analysis and identify the significantly differential genes after enhanced expression of KDM3B. After bioinformatic analysis, the GO analysis indicated that some upregulated GO functions might be linked to osteo-/odontogenic differentiation, cell proliferation, and migration potential including positive regulation of cell migration, positive regulation of cell motility, positive regulation of cellular component movement, and positive regulation of response to external stimulus. Then, the Path-net analysis results suggest that the Janus kinase signal transducer and activator of transcription (JAK-STAT) and Toll-like receptor signaling pathways may be involved in the functional regulation of KDM3B. At present, some researches have shown that the JAK-STAT pathway can modulate cell migration potentials of BMSCs [40] while regulating bone regeneration and promoting angiogenesis [41–43]. Among them, the STAT1-CCND1/CDK6 axis promotes proliferation by accelerating the transition from the G0/G1 phase to the S phase [44, 45]. These genes are believed to be key factors in cell proliferation. Besides, it is generally known that MSCs are recruited to the injured or inflamed sites through TLR receptor-mediated interactions to promote tissue repair and regeneration [46]. It has been confirmed that potent agonists of TLR2, TLR3, and TLR4 enhanced the ability of MSCs to differentiate into osteoblasts [47]. As a key receptor of TLRs, MYD88 can enhance the expression of RUNX2 and ALP activity induced by TLR4 [48]. MSCs isolated from Myd88-deficient mice cannot differentiate into osteoblasts [49]. This indicates that KDM3B may enhance the osteo-/odontogenic differentiation ability of SCAPs by promoting the expression of MYD88, but further study is needed. Meanwhile, other core genes also play an important role in regulating the function of MSCs. FGF5 promotes osteogenic differentiation and cell proliferation potential of BMSCs by activating ERK1/2 [50]. Proteolytic enzymes, such as PLAU, may play a crucial role in cell migration and tissue remodeling during tissue regeneration. However, SERPINE1 can reverse the function of PLAU as an inhibitor of PLAU [51]. In general, our findings confirmed that KDM3B may affect the biological function of SCAPs by regulating some candidate downstream genes, but the molecular mechanism and regulation function remain to be further studied.

## 5. Conclusions

In conclusion, we identified that KDM3B promoted the osteo-/odontogenic differentiation, cell proliferation, and migration potential of SCAPs. We also identified the key downstream genes and possible pathways of KDM3B involved in these processes. These discoveries contribute to the understanding of the function and mechanism of KDM3B and provide a novel target for the regulation of MSCs and promoted MSC-mediated bone and tooth regeneration.

## Figures and Tables

**Table 1:** Primer sequences used in the real-time RT-PCR.

Gene symbol	Primer sequences (5′-3′)
*GAPDH*-F	CGGACCAATACGACCAAATCCG
*GAPDH*-R	AGCCACATCGCTCAGACACC
*RUNX2*-F	TCTTAGAACAAATTCTGCCCTTT
*RUNX2*-R	TGCTTTGGTCTTGAAATCACA
*OSX*-F	CCTCCTCAGCTCACCTTCTC
*OSX*-R	GTTGGGAGCCCAAATAGAAA

## Data Availability

The data used to support the findings of this study are included within the article.

## Supplementary Materials

Supplementary 1 Figure 1:The cell image under the microscope

Supplementary 2 Table 1:The differentially expressed genes in KDM3B overexpressed SCAPs compared with control group

Supplementary 3 Table 2:The upregulated and downregulated pathways in KDM3B over-expressed SCAPs

Supplementary 4 Table 3:The significant pathways enriched by path-net in KDM3B over-expressed SCAPs

Supplementary 5 Table 4:The 12 core genes enriched by signal-net in KDM3B over-expressed SCAPs
