# Supplementary figures and images for: The Histone Demethylase KDM3B Promotes Osteo-/Odontogenic Differentiation, Cell Proliferation, and Migration Potential of Stem Cells from the Apical Papilla
Source: Stem Cells Int. 2020 Oct 7;2020:8881021. doi: 10.1155/2020/8881021 (PMC7563049; doi:10.1155/2020/8881021)

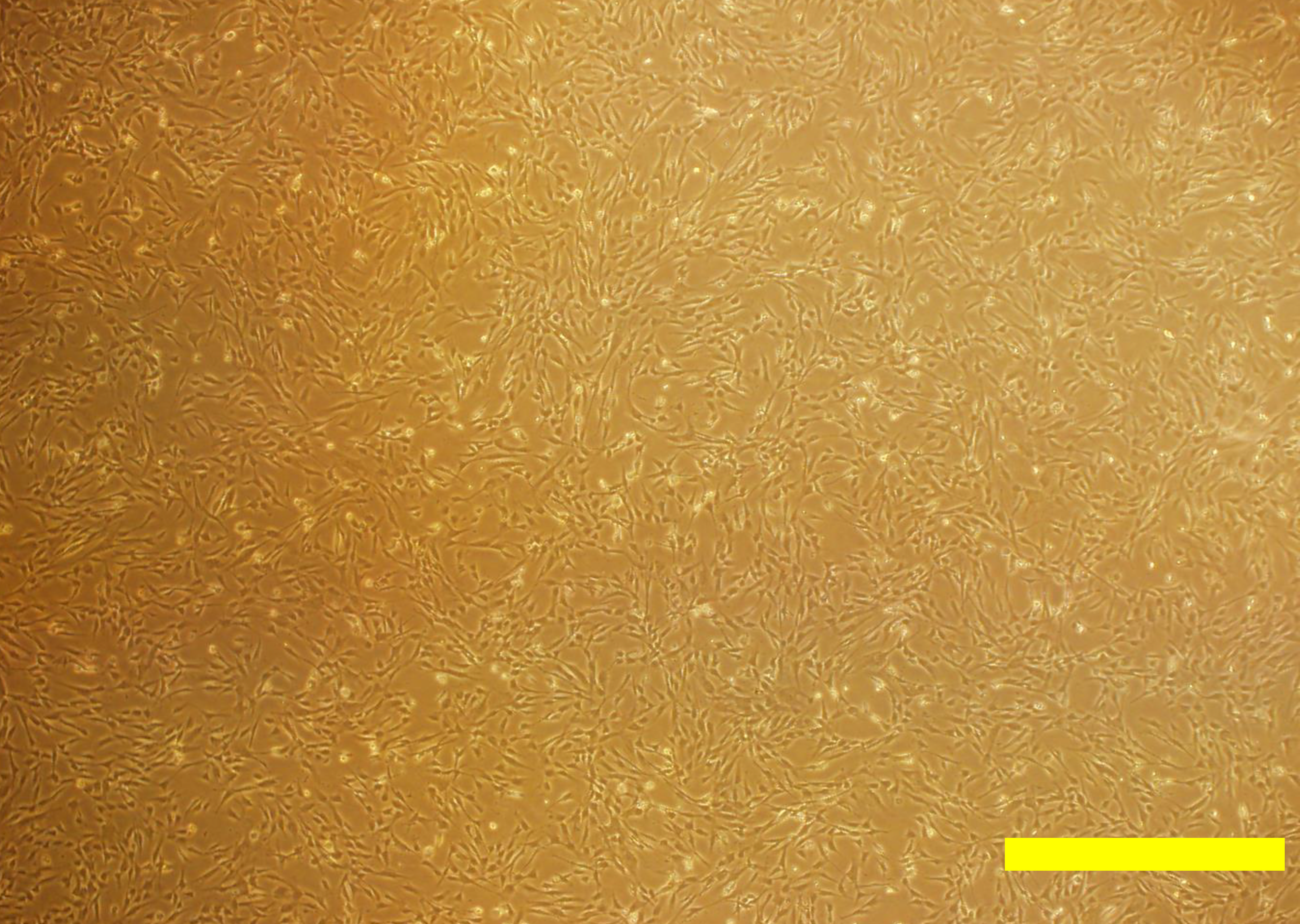

Supplement: Supplementary 1 — Figure 1:The cell image under the microscope [file 8881021.f1.tiff]
