# Supplementary material for: The Histone Demethylase KDM3B Promotes Osteo-/Odontogenic Differentiation, Cell Proliferation, and Migration Potential of Stem Cells from the Apical Papilla
Source: Stem Cells Int. 2020 Oct 7;2020:8881021. doi: 10.1155/2020/8881021 (PMC7563049; doi:10.1155/2020/8881021)
Supplement: Supplementary 2 — Table 1:The differentially expressed genes in KDM3B overexpressed SCAPs compared with control group [file 8881021.f2.docx]

**Supplementary Table 1. The differentially expressed genes in KDM3B overexpressed SCAPs compared with control group**

| Gene Symbol | mRNA_Accession | | Fold Change | P.Value | diffState | |
| --- | --- | --- | --- | --- | --- | --- |
| RSAD2 | | NM_080657 | 4.295699563 | 8.56305E-06 | | up |
| IFIT2 | | NM_001547 | 2.92481597 | 2.95326E-05 | | up |
| MX2 | | NM_002463 | 2.886567229 | 1.64797E-05 | | up |
| MX1 | | NM_001144925 | 2.827937845 | 1.85309E-05 | | up |
| IFIT3 | | NM_001031683 | 2.504703864 | 4.3127E-05 | | up |
| OAS1 | | NM_001032409 | 2.431134904 | 2.20308E-05 | | up |
| OAS3 | | NM_006187 | 2.333690381 | 8.5469E-05 | | up |
| OAS2 | | NM_001032731 | 2.289408164 | 1.40993E-05 | | up |
| ANGPTL4 | | NM_001039667 | 2.280666687 | 0.000166895 | | up |
| TGM2 | | NM_004613 | 2.256225107 | 8.19085E-06 | | up |
| HERC6 | | NM_001165136 | 2.106418533 | 2.30631E-05 | | up |
| BST2 | | NM_004335 | 2.091805579 | 5.95623E-05 | | up |
| DDX58 | | NM_014314 | 2.071173357 | 7.93219E-05 | | up |
| SAMD9 | | NM_001193307 | 2.059148985 | 8.62562E-05 | | up |
| TLR3 | | NM_003265 | 2.053285283 | 5.68397E-06 | | up |
| ITGA2 | | NM_002203 | 1.999073033 | 9.99256E-06 | | up |
| DDX60L | | NM_001012967 | 1.996643874 | 1.17855E-05 | | up |
| SAMD9L | | NM_001303496 | 1.989392338 | 4.2294E-05 | | up |
| PARP12 | | NM_022750 | 1.98642271 | 2.3556E-05 | | up |
| IFIT1 | | NM_001270927 | 1.980496736 | 7.44462E-05 | | up |
| IL7R | | NM_002185 | 1.968457686 | 2.48924E-05 | | up |
| PLAT | | NM_000930 | 1.948208412 | 9.18437E-05 | | up |
| PARP14 | | NM_017554 | 1.879608877 | 5.74943E-05 | | up |
| STC1 | | NM_003155 | 1.87829592 | 0.000193146 | | up |
| EVI2B | | NM_006495 | 1.860079241 | 0.000609304 | | up |
| SEMA7A | | NM_001146029 | 1.851880593 | 3.89833E-05 | | up |
| OASL | | NM_001261825 | 1.827144959 | 0.000342675 | | up |
| USP18 | | NM_017414 | 1.808672669 | 0.000275651 | | up |
| IFI44L | | NM_006820 | 1.805987906 | 1.29433E-05 | | up |
| SP110 | | NM_001185015 | 1.800383904 | 0.000288153 | | up |
| IFIH1 | | NM_022168 | 1.788649937 | 0.000498801 | | up |
| TAP2 | | NM_000544 | 1.761504309 | 1.64093E-05 | | up |
| TM4SF1 | | NM_014220 | 1.735562185 | 0.003121668 | | up |
| TRIM21 | | NM_003141 | 1.734535026 | 0.001251562 | | up |
| ZNFX1 | | NM_021035 | 1.730735881 | 5.22124E-05 | | up |
| XAF1 | | NM_017523 | 1.723336269 | 2.2732E-05 | | up |
| CMPK2 | | NM_001256477 | 1.722858921 | 2.05856E-05 | | up |
| DUSP6 | | NM_001946 | 1.722061305 | 0.001502709 | | up |
| TRIM14 | | NM_014788 | 1.720368609 | 0.001719087 | | up |
| NAV3 | | NM_001024383 | 1.718301545 | 0.004136164 | | up |
| FGF5 | | NM_001291812 | 1.708243122 | 0.009074155 | | up |
| NCEH1 | | NM_001146276 | 1.707221337 | 0.001076278 | | up |
| KRTAP1-5 | | NM_031957 | 1.701208302 | 0.001828599 | | up |
| GPX3 | | NM_002084 | 1.698183001 | 2.67887E-05 | | up |
| PARP9 | | NM_001146102 | 1.684659741 | 0.000156545 | | up |
| RNF152 | | NM_173557 | 1.684647644 | 0.000709675 | | up |
| USP53 | | NM_019050 | 1.683990189 | 0.000467974 | | up |
| SERINC2 | | NM_001199037 | 1.682458304 | 0.000171219 | | up |
| SPRY4 | | NM_001127496 | 1.68072857 | 0.004862877 | | up |
| PDCD1LG2 | | NM_025239 | 1.679782417 | 0.012839222 | | up |
| CYGB | | NM_134268 | 1.677816833 | 6.75025E-05 | | up |
| MYD88 | | NM_001172566 | 1.673301782 | 0.000166411 | | up |
| SLC16A6 | | NM_001174166 | 1.67199223 | 0.004087842 | | up |
| MYPN | | NM_001256267 | 1.647490064 | 0.008400814 | | up |
| SEMA3A | | NM_006080 | 1.643661131 | 0.002077504 | | up |
| BCL2L1 | | NM_001191 | 1.64118707 | 0.001512882 | | up |
| CCND1 | | NM_053056 | 1.63581777 | 6.54129E-05 | | up |
| SLC14A1 | | NM_001128588 | 1.634048578 | 0.001897775 | | up |
| FJX1 | | NM_014344 | 1.630251448 | 0.000295759 | | up |
| ITGA3 | | NM_002204 | 1.626487835 | 0.000227633 | | up |
| ANKRD1 | | NM_014391 | 1.618467255 | 5.86122E-05 | | up |
| CLDN11 | | NM_001185056 | 1.617444632 | 0.000113772 | | up |
| DTX3L | | NM_138287 | 1.6173602 | 0.001324562 | | up |
| PNPT1 | | NM_033109 | 1.614743812 | 0.013344627 | | up |
| GMPR | | NM_006877 | 1.613555453 | 4.45974E-05 | | up |
| IFI30 | | NM_006332 | 1.612730945 | 0.000281596 | | up |
| CLDN1 | | NM_021101 | 1.610767387 | 0.000661131 | | up |
| SLC2A1 | | NM_006516 | 1.600984534 | 0.000179454 | | up |
| SLC8A1 | | NM_001112800 | 1.59791238 | 0.000759948 | | up |
| CHRNA1 | | NM_000079 | 1.59378758 | 0.000610438 | | up |
| NPAS2 | | NM_002518 | 1.590192479 | 0.001737594 | | up |
| MET | | NM_000245 | 1.587940303 | 0.016866461 | | up |
| SERPINE1 | | NM_000602 | 1.587871856 | 0.000252557 | | up |
| C19orf66 | | NM_001308277 | 1.578440702 | 4.95281E-05 | | up |
| PPRC1 | | NM_001288727 | 1.570307586 | 0.000554874 | | up |
| EIF2AK2 | | NM_001135651 | 1.565908818 | 0.000438227 | | up |
| SAMHD1 | | NM_015474 | 1.560508288 | 0.00029231 | | up |
| MMP3 | | NM_002422 | 1.557165387 | 0.000780652 | | up |
| SLC43A3 | | NM_001278201 | 1.555113826 | 0.003067513 | | up |
| FOSL1 | | NM_001300844 | 1.544929299 | 0.008766638 | | up |
| CNIH3 | | NM_152495 | 1.543836678 | 0.011074941 | | up |
| DDX60 | | NM_017631 | 1.536266844 | 0.001778019 | | up |
| RGMB | | NM_001012761 | 1.533865644 | 0.007016335 | | up |
| DCBLD2 | | NM_080927 | 1.531292656 | 0.001773596 | | up |
| PLAU | | NM_001145031 | 1.529640845 | 0.000355759 | | up |
| NM_030754.2 | | NM_030754.2 | 1.523701651 | 0.041751748 | | up |
| IFI44 | | NM_006417 | 1.521858762 | 0.000256363 | | up |
| MGLL | | NM_001003794 | 1.517490998 | 0.002126144 | | up |
| UBA7 | | NM_003335 | 1.517037894 | 0.000566331 | | up |
| SLC15A3 | | NM_016582 | 1.515971375 | 0.000225492 | | up |
| TRIM22 | | NM_001199573 | 1.512839828 | 0.000748273 | | up |
| DDAH1 | | NM_001134445 | 1.51120594 | 0.001459247 | | up |
| STAT1 | | NM_007315 | 1.510360509 | 7.05353E-05 | | up |
| MYADM | | NM_001020818 | 1.50950308 | 0.001026715 | | up |
| IFI27 | | NM_001130080 | 1.509343245 | 0.011213502 | | up |
| CTGF | | NM_001901 | 1.508669076 | 0.001539317 | | up |
| ISG15 | | NM_005101 | 1.508408534 | 0.004250361 | | up |
| AGRN | | NM_001305275 | 1.507756515 | 8.29449E-05 | | up |
| PCMTD1 | | NM_001286782 | 0.666105847 | 0.000121758 | | down |
| WDR27 | | NM_001202550 | 0.666023994 | 0.002394494 | | down |
| SAT2 | | NM_133491 | 0.663911403 | 0.000748372 | | down |
| POLR3GL | | NM_032305 | 0.663509252 | 0.002496222 | | down |
| LAMA4 | | NM_001105206 | 0.661287609 | 0.000105131 | | down |
| CTSK | | NM_000396 | 0.659914818 | 0.000499508 | | down |
| C10orf10 | | NM_007021 | 0.656529321 | 0.001837154 | | down |
| RPS12 | | NM_001016 | 0.655024597 | 0.002888347 | | down |
| PSAT1 | | NM_021154 | 0.652729559 | 0.00178048 | | down |
| THBS2 | | NM_003247 | 0.65141938 | 0.000150465 | | down |
| RBP1 | | NM_001130992 | 0.650068043 | 7.79688E-05 | | down |
| VAT1L | | NM_020927 | 0.648647729 | 0.001324047 | | down |
| ASNS | | NM_001178075 | 0.645141334 | 0.000557137 | | down |
| AMPH | | NM_001635 | 0.643686632 | 0.000525039 | | down |
| NUPR1 | | NM_001042483 | 0.64335632 | 0.000300377 | | down |
| KLHL24 | | NM_017644 | 0.638798187 | 0.001135228 | | down |
| LUM | | NM_002345 | 0.638003888 | 0.000535065 | | down |
| ID3 | | NM_002167 | 0.63453265 | 0.002204498 | | down |
| TSC22D3 | | NM_001015881 | 0.632060806 | 0.000539865 | | down |
| LHX8 | | NM_001001933 | 0.62850483 | 0.0001704 | | down |
| RPS13 | | NM_001017 | 0.624764001 | 0.00774283 | | down |
| ARHGEF19 | | NM_153213 | 0.622900197 | 0.001468789 | | down |
| ITGA8 | | NM_001291494 | 0.622833518 | 0.000164946 | | down |
| COL3A1 | | NM_000090 | 0.62163473 | 0.001203232 | | down |
| DEPTOR | | NM_001283012 | 0.620684903 | 0.002465409 | | down |
| ITGB8 | | NM_002214 | 0.610196382 | 0.003477972 | | down |
| SEPP1 | | NM_001085486 | 0.609493042 | 0.000343092 | | down |
| SNED1 | | NM_001080437 | 0.594931284 | 0.001793079 | | down |
| VCAM1 | | NM_001078 | 0.593735441 | 4.78144E-05 | | down |
| SESN3 | | NM_001271594 | 0.593697996 | 0.004348846 | | down |
| SAT1 | | NM_002970 | 0.590320434 | 0.000545707 | | down |
| SERPINA3 | | NM_001085 | 0.589448681 | 0.000307374 | | down |
| C5AR2 | | NM_001271749 | 0.583489776 | 0.000499506 | | down |
| TMEM119 | | NM_181724 | 0.580147839 | 6.13964E-05 | | down |
| PENK | | NM_001135690 | 0.578228313 | 1.84024E-05 | | down |
| COX7B | | NM_001866 | 0.571386784 | 0.02701008 | | down |
| GPNMB | | NM_001005340 | 0.554726645 | 0.000150124 | | down |
| SNCAIP | | NM_001242935 | 0.540556543 | 0.008549281 | | down |
| SERPINF1 | | NM_002615 | 0.538514625 | 2.72434E-05 | | down |
| CXCL12 | | NM_000609 | 0.53795617 | 0.001751115 | | down |
| HSD17B14 | | NM_016246 | 0.528585224 | 2.73999E-05 | | down |
| FGF7 | | NM_002009 | 0.526367418 | 0.000405623 | | down |
| MFAP4 | | NM_001198695 | 0.518692719 | 3.62091E-05 | | down |
| POSTN | | NM_001135934 | 0.502143093 | 1.80967E-06 | | down |
| EDNRB | | NM_000115 | 0.492515476 | 6.92754E-05 | | down |
| DIO2 | | NM_000793 | 0.466900916 | 0.004200376 | | down |
| ID2 | | NM_002166 | 0.439582981 | 4.11975E-06 | | down |
| OMD | | NM_005014 | 0.409186533 | 1.05809E-06 | | down |
